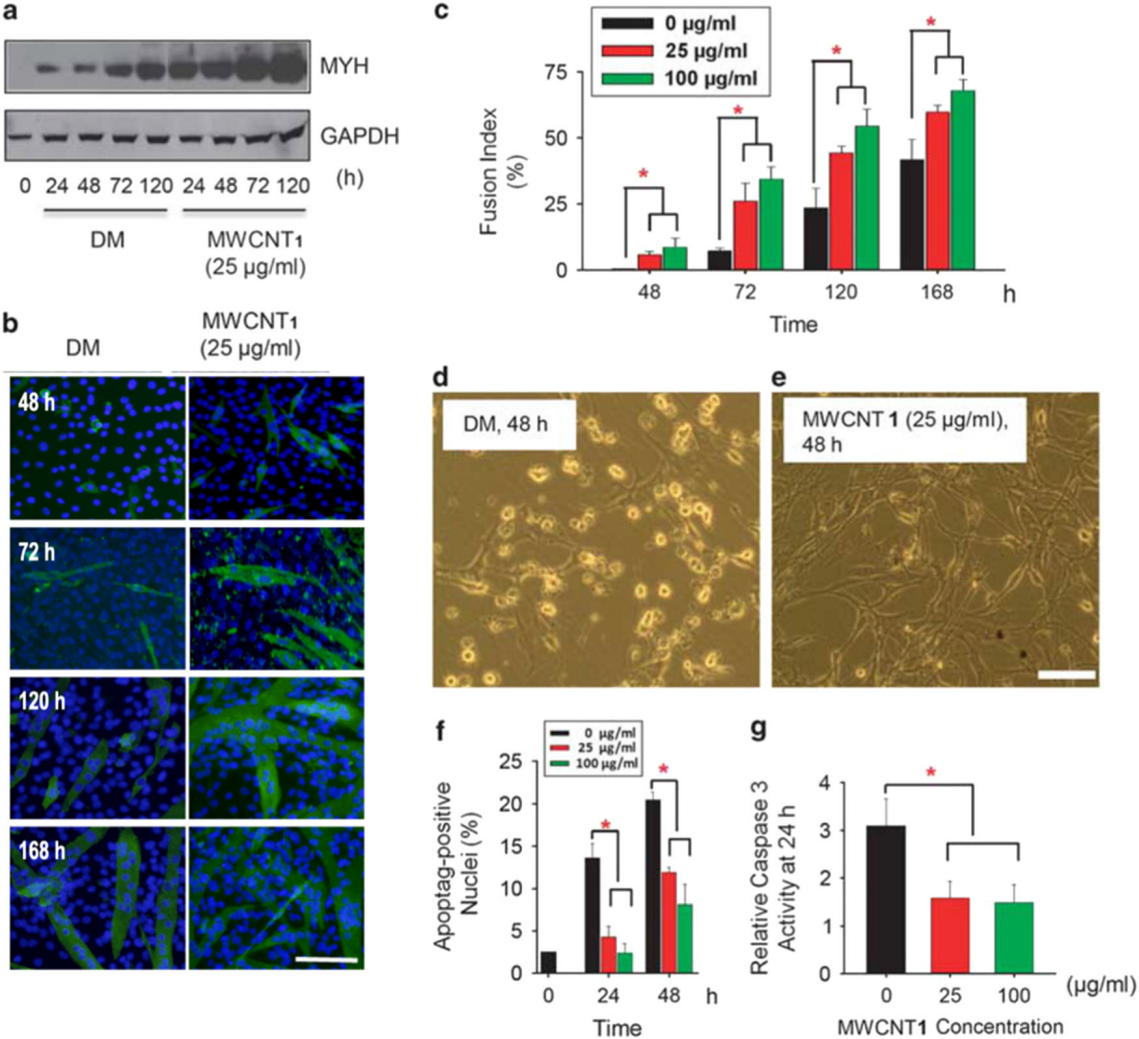# Correction to: Binding of carbon nanotube to BMP receptor 2 enhances cell differentiation and inhibits apoptosis via regulating bHLH transcription factors

**DOI:** 10.1038/s41419-024-07291-4

**Published:** 2025-01-21

**Authors:** Y. Zhang, Q. Mu, H. Zhou, K. Vrijens, M. F. Roussel, G. Jiang, B. Yan

**Affiliations:** 1 School of Pharmaceutical Sciences, Shandong University, Jinan, China; 2 Department of Chemical Biology and Therapeutics, St. Jude Children’s Research Hospital, Memphis, TN USA; 3 Department of Tumor Cell Biology, St. Jude Children’s Research Hospital, Memphis, TN USA; 4 Research Center for Eco-Environmental Sciences, Chinese Academy of Sciences, Beijing, China; 5 School of Chemistry and Chemical Engineering, Shandong University, Jinan, China

**Correction to:** *Cell Death & Disease* 10.1038/cddis.2012.48, published online 10 May 2012

In Fig. 1b, the image of 72 h MWCNT1 (25 μg/ml) was incorrectly used. The following figure should replace the original Fig. 1. This change does not affect the reported results, discussions, or conclusions in this paper. We apologize for this oversight.